# Phytoplankton responses to dust addition in the Fe–Mn co-limited eastern Pacific sub-Antarctic differ by source region

**DOI:** 10.1073/pnas.2220111120

**Published:** 2023-07-03

**Authors:** Neil J. Wyatt, Antony Birchill, Simon Ussher, Angela Milne, Heather A. Bouman, Elizabeth Shoenfelt Troein, Katsiaryna Pabortsava, Alan Wright, Oliver Flanagan, Thomas S. Bibby, Adrian Martin, C. Mark Moore

**Affiliations:** ^a^School of Ocean and Earth Science, National Oceanography Centre Southampton, University of Southampton, Southampton SO14 3ZH, United Kingdom; ^b^School of Geography, Earth and Environmental Sciences, University of Plymouth, Plymouth PL4 8AA, United Kingdom; ^c^Department of Earth Sciences, University of Oxford, Oxford OX1 2JD, United Kingdom; ^d^Department of Earth, Atmospheric and Planetary Sciences, Massachusetts Institute of Technology, Cambridge, MA 02139; ^e^National Oceanography Centre, Southampton SO14 3ZH, United Kingdom

**Keywords:** biogeochemistry, phytoplankton, ecophysiology

## Abstract

The seasonal availability of light and micronutrients strongly regulates productivity in the Southern Ocean, restricting biological utilization of macronutrients and CO_2_ drawdown. Mineral dust flux is a key conduit for micronutrients to the Southern Ocean and a critical mediator of multimillennial-scale atmospheric CO_2_ oscillations. While the role of dust-borne iron (Fe) in Southern Ocean biogeochemistry has been examined in detail, manganese (Mn) availability is also emerging as a potential driver of past, present, and future Southern Ocean biogeochemistry. Here, we present results from fifteen bioassay experiments along a north–south transect in the undersampled eastern Pacific sub-Antarctic zone. In addition to widespread Fe limitation of phytoplankton photochemical efficiency, we found further responses following the addition of Mn at our southerly stations, supporting the importance of Fe–Mn co-limitation in the Southern Ocean. Moreover, addition of different Patagonian dusts resulted in enhanced photochemical efficiency with differential responses linked to source region dust characteristics in terms of relative Fe/Mn solubility. Changes in the relative magnitude of dust deposition, combined with source region mineralogy, could hence determine whether Fe or Mn limitation control Southern Ocean productivity under future as well as past climate states.

## Significance

Atmospheric dust supply of micronutrients to the Southern Ocean is an important regulator of phytoplankton productivity, impacting the global carbon cycle. Understanding how phytoplankton respond to changes in dust supply has become increasingly important, with supply predicted to alter with future climate change. We show that Patagonian dusts, which differ in characteristics by source region, can supply different amounts of the essential micronutrients iron and manganese to the Southern Ocean and subsequently drive different responses in the resident phytoplankton. Changes in dust supply, including shifts in source regions, could therefore be an important factor controlling phytoplankton productivity in the past and future Southern Ocean.

Productivity in the modern Southern Ocean is restricted by low iron (Fe) and potentially manganese (Mn) availability (1–4) owing to upwelling of macronutrient-rich deep waters depleted in these scavenged trace metals (5, 6), combined with low atmospheric supply (1, 7).

Both Fe and Mn are essential requirements in oxygenic photosynthesis, with multiple Fe-binding components required to enable photosynthetic electron flow, while the oxygen-evolving complex of photosystem II requires 4 bound Mn atoms (8). Surface uptake by phytoplankton leads to a scarcity of both Fe and Mn in Southern Ocean surface waters, which can subsequently limit productivity, leading to underutilization of macronutrients and net release of deep-ocean carbon (4, 7, 9). Enhanced micronutrient supply, including through increased atmospheric dust fluxes, thus has the potential to drive increased macronutrient utilization, export production, and atmospheric CO_2_ drawdown (1, 7). Indeed, the higher supply of lithogenic Fe to Southern Ocean phytoplankton during glacial climate periods correlates with increased nitrate removal and productivity and is a critical mediator of millennial-scale atmospheric CO_2_ oscillations (7, 10–12). Dust fluxes to the sub-Antarctic zone (SAZ) in particular have been associated with a significant fraction of atmospheric CO_2_ drawdown during the last glacial cycle (9, 13), with isolation of the deep Southern Ocean by enhanced stratification being the other major contributor (13–15).

Although there is still uncertainty as to whether global dust deposition rates will increase or decrease due to anthropogenic climate change (16, 17), the expansion of deserts could result in a threefold greater atmospheric dust loading by the end of the 21st century (18). Regionally, this expansion, coupled with climate-related increased storminess, could result in a tenfold increase in dust loading over the Southern Hemisphere compared with a minimal increase over much of the Northern Hemisphere (18). Any future increase in dust flux may enhance the delivery of Fe and other co-limiting nutrients such as Mn (4, 19), which could be particularly important should warming and freshening of Southern Ocean surface waters increase stratification, and hence reduce supply from subsurface reservoirs (5, 20). The delivery of dust-borne nutrients may be further supported by an increased contribution from wildfire aerosols (21–24), which can relieve nutrient limitation and significantly increase Southern Ocean productivity following exceptional individual events (25).

Despite the potential importance of Southern Ocean dust inputs for modern productivity and glacial–interglacial cycles, relatively few studies have investigated the ecophysiological response of natural Southern Ocean phytoplankton communities to direct dust addition. In situ and shipboard bottle experiments of Fe fertilization have typically assessed responses to the addition of inorganic Fe (e.g., dissolved chloride salts or ferrous sulfate) (26–30). Such addition of Fe may fail to mimic the responses from complex natural substrates, where the bioavailability of multiple micronutrients may vary. For example, naturally Fe-limited Southern Ocean phytoplankton have been shown to respond differently to dust addition compared with inorganic Fe (31). Moreover, the increasing evidence that Mn may be co-limiting with Fe in certain settings and regions of the Southern Ocean (4, 19, 32) needs consideration in the context of multiple-micronutrient supply from natural particulates. This is particularly interesting due to the high abundance of both elements in crustal material (sediments, airborne dusts) and similar short oceanic residence times. Indeed, the supply of volcanic ash to Southern Ocean phytoplankton communities has been shown to produce a stronger response than addition of Fe alone, potentially due to relief of Mn (co-)limitation (33). Understanding biological responses to complex natural sources of trace metal nutrients may thus be essential for realistic projections of the Southern Ocean biological carbon pump under past and future climates (4).

The composition of dust will vary with source provenance (34, 35) and processing both at source and during atmospheric transport (36, 37). In particular, Fe within glaciogenic versus nonglaciogenic dust sources appears to be both more labile and bioavailable due to a higher Fe^2+^ content (35, 38). This higher Fe^2+^ content of glaciogenic dust has been shown to significantly increase growth and photosynthetic efficiency compared with nonglaciogenic dust in a cultured diatom (35). Similarly, the relative abundance, solubility, and bioavailability of the multiple trace metals in dust deposition may be key in determining the overall ecosystem response (4).

South America is the largest source of dust to the modern Southern Ocean, contributing ~60% of the flux, with Australia the other key source, particularly for the Pacific sector (39, 40). However, increased circumpolar transport of South American dust sources potentially dominated the >threefold higher dust fluxes and >15-fold higher Fe^2+^ fluxes to the Pacific SAZ during glacial periods (34, 41). Understanding the fertilization potential of this increased glacial bioavailable Fe^2+^ flux, alongside other potentially co-limiting trace metals for natural phytoplankton communities in the Pacific SAZ, may thus be crucial for determining the likely response of the glacial–interglacial system and more broadly understanding the role of variable dust fluxes and source regions in Southern Ocean biogeochemistry.

**Fig. 1.**
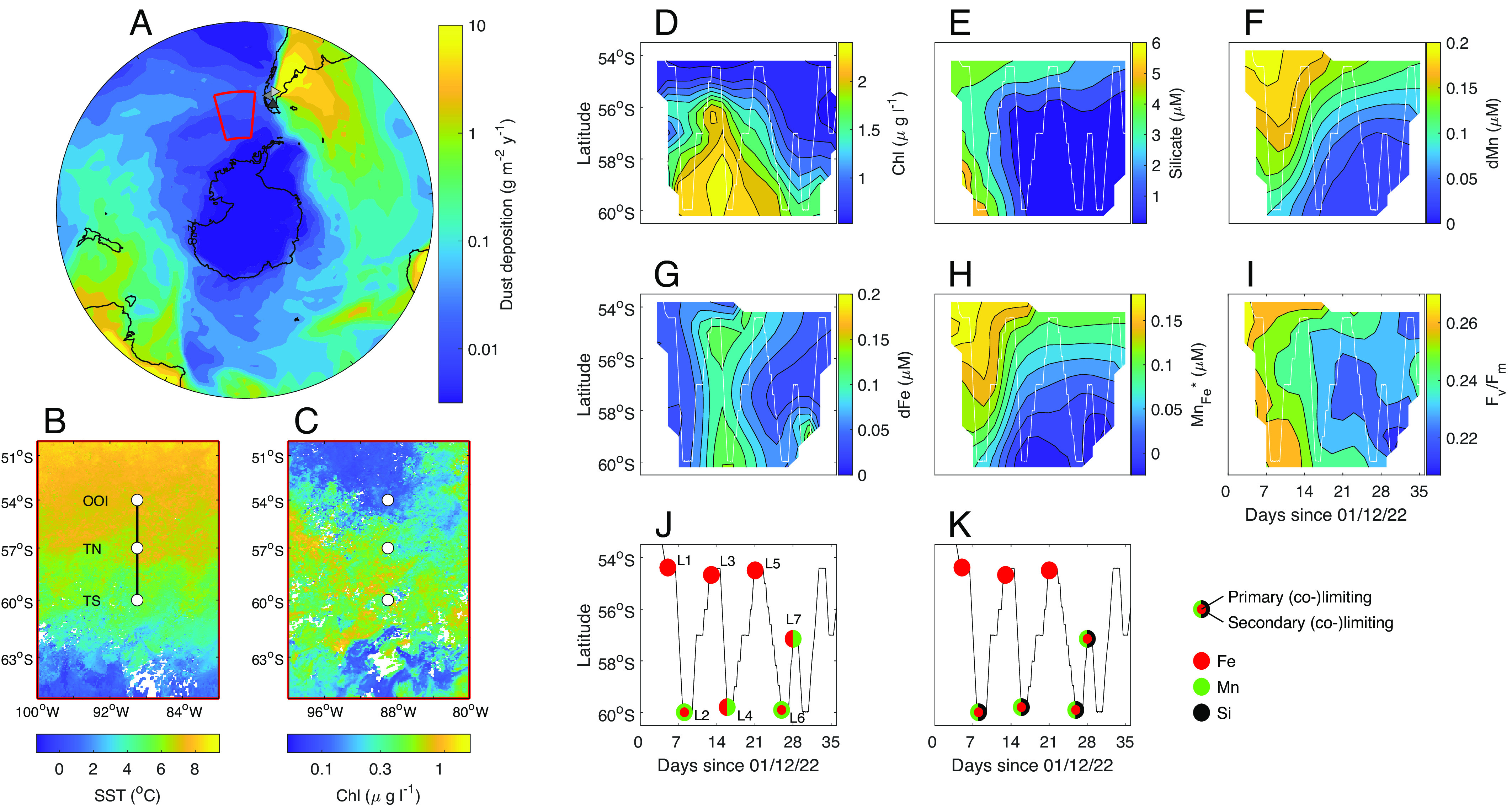
Biogeochemical setting and spatiotemporal development of Fe and Mn (co-)limitation. (*A*) Annual mean dust deposition (g m^−2^ y^−1^) in and around the Southern Ocean (24) alongside (*B*) December 2019 mean sea surface temperature and (*C*) chlorophyll-*a* concentration (Modis AQUA at 4 km resolution) for the study region (red trapezoid in 1A). (*D*–*I*) Hovmöller diagrams (time against latitude) showing in situ biogeochemical progression during the DY111 cruise for sea surface chlorophyll-*a* (*D*), silicate (*E*), dMn (*F*), dFe (*G*), Mn_Fe_* (*H*), *F_v _/F_m_* (*I*). (*J* and *K*) Summarized experimental responses (large-volume experiments after 6 d) to nutrient addition as indicated by changes in *F_v_ /F_m_* (*J*) and chlorophyll-*a* (*K*). Symbol colors indicate the identity and type of limitation, see *SI Appendix*, Figs. S1–S3 (42). Locations of the large-type experiments are labeled in *J*, see *SI Appendix*, Fig. S6 for all experimental times/locations.

We thus combined in situ observations with a series of shipboard bottle experiments in the undersampled eastern Pacific SAZ (3), the region of the modern sub-Antarctic with the lowest annual mean dust flux (Fig. 1*A*). Experiments were conducted to assess the potential role of natural sources of Fe and Mn in regulating phytoplankton ecophysiology in the region and the broader Southern Ocean.

## Results and Discussion

### Phytoplankton Responses to Fe and Mn Amendment.

Eight smaller-volume (2 to 6 d, denoted Ex-S1-S8) and seven larger-volume (6 d, denoted Ex-L1-L7) nutrient addition experiments were conducted using various factorial combinations of inorganic Fe and Mn [alongside silicate (Si) and zinc (Zn)], in addition to three different Patagonian dust sources (35) (*Materials and Methods*). Phytoplankton responses to amendment with Fe and/or Mn showed clear spatiotemporal patterns coincident with in situ variability in the concentration of these metals and prevailing phytoplankton bloom conditions, as influenced by physical setting (Fig. 1*B*) and indicated by macronutrient drawdown and chlorophyll-*a* concentrations (Fig. 1 *C*–*E*). Surface chlorophyll-*a* concentrations (Fig. 1 *C* and *D*) indicated a substantial bloom peaking shortly after the start of the cruise in the southern half of the study region. Correspondingly, surface dissolved silicate (dSi) and Mn (dMn) concentrations (Fig. 1 *E* and *F*) decreased over time as the bloom progressed, particularly to the south of the region. In contrast, dissolved Fe (dFe) concentrations (Fig. 1*G*) were more uniformly low (<0.1 nmol L^−1^) in surface waters over the observation period.

**Fig. 2.**
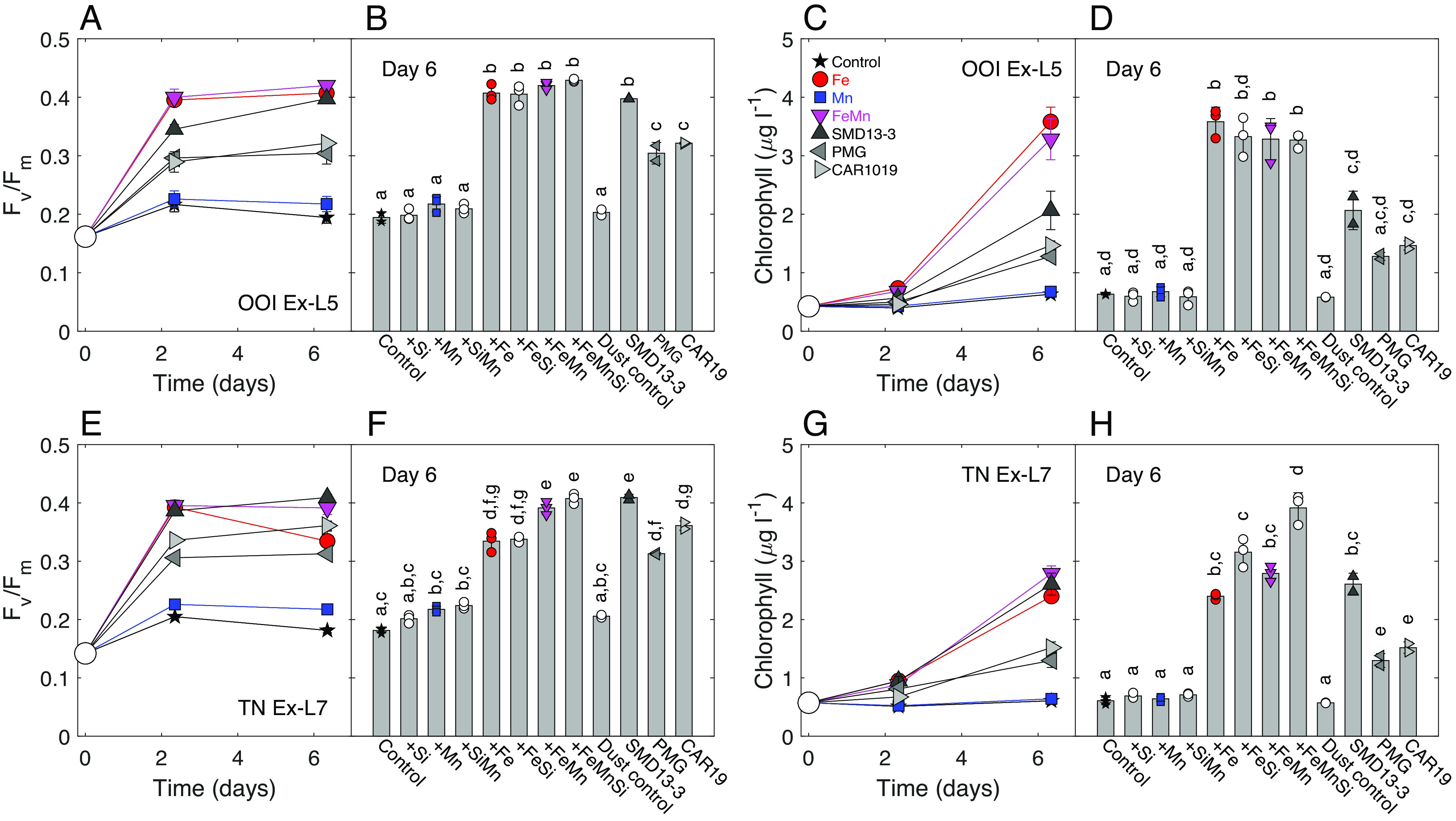
Example responses of phytoplankton ecophysiology to nutrient and dust additions. (*A*, *B*, *E*, and *F*) Apparent photochemical efficiency of PSII (*F_v_ /F_m_*) and (*C*, *D*, *G*, and *H*) chlorophyll-*a* response to nutrient and dust amendment in Ex-L5 at OOI (*A*–*D*) and Ex-L7 at TN (*E*–*H*), respectively. For clarity, only a subset of the nutrient and dust additions are shown in the time series *A*, *C*, *E*, and *G*, with the day 6 data for all treatments included in *B*, *D*, *F*, and *H*. Symbols for all panels are indicated in (*C*). Large open circles in *A*, *C*, *E*, and *G* indicate initial values. Means (±1 SD) are indicated in all panels, with individual data points also provided (small symbols) for *B*, *D*, *F*, and *H*. Statistically indistinguishable means evaluated across all treatments in full factorial manner are labeled with the same letter (ANOVA followed by Bonferroni post-hoc mean comparison test *P* ≤ 0.05).

The supply of Fe to incubation experiments significantly increased values of the fluorescence-derived parameter *F_v_  /F_m_* in all experiments (Fig. 2 and *SI Appendix*, Figs. S1, S4, and S6), indicating widespread Fe limitation of the efficiency with which extant phytoplankton were converting absorbed light energy to chemical energy during photosynthesis (43). No serial responses [i.e., greater secondary or tertiary effects following the addition of a second or third nutrient in combination, see *Materials and Methods* (42)] were observed at the lower biomass northern OOI station, where dMn remained relatively high (dMn > 0.1 nmol L^−1^) (Figs. 1 and 2 and *SI Appendix*, Figs. S1–S6). In contrast, at stations TN and TS, toward the south of the SAZ, which were characterized by elevated chlorophyll-*a* and rapidly depleting dMn and dSi concentrations (Fig. 1 *E* and *F*), phytoplankton experimental responses indicated Mn serial/co-stress within all experiments, i.e., *F_v_ /F_m_* was significantly increased following combined Fe and Mn addition compared with Fe alone [irrespective of further addition of Si (i.e., ±Si)]. Importantly, the observed significant differences in *F_v_ /F_m_* between Fe(±Si) and FeMn(±Si) treatments were characterized by a drop in *F_v_/F_m_* within Fe(±Si) treatments from day 2 to day 6 (e.g., Fig. 2*E*). Moreover, a small but statistically significant increase in *F_v_ /F_m_* following sole addition of Mn(±Si) was observable after 6 d at TS in Ex-L4 and in TN Ex-L7 (Fig. 2*F* and *SI Appendix*, Fig. S1).

**Fig. 3.**
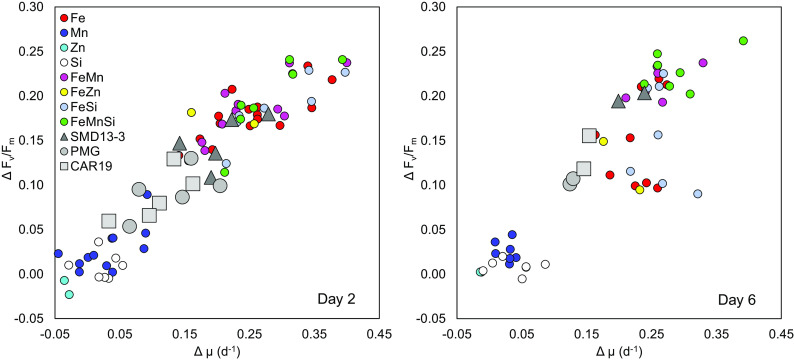
Change in apparent photochemical efficiency of PSII (Δ*F_v _/F_m_*) and net growth rates (µ) in response to nutrient and dust amendment across all experiments. Delta notation (Δ) indicates change relative to the values in control bottles. Data shown here correspond to the mean responses (*n* = 2 to 3) from all treatments across all bioassay experiments.

Chlorophyll-*a* concentrations (Fig. 2 and *SI Appendix*, Figs. S2 and S5) and DIN drawdown (*SI Appendix*, Fig. S3) increased alongside *F_v_ /F_m_* following Fe addition to all experiments. Serial responses to the further addition of Mn and/or Si were always observed to the south of the study region at TN and TS, with the highest chlorophyll-*a* increases and DIN drawdown occurring following the combined FeMnSi addition to these experiments, despite no evidence of primary Si limitation. Our experiments thus indicated Fe–Mn–Si serial limitation of the phytoplankton community in the south of our study region. In contrast to Fe and Mn, that have an absolute requirement in photosynthesis (8, 19), no change in *F_v_ /F_m_* or biomass was observed following the addition of Zn within the two experiments where this was tested (Fig. 3 and *SI Appendix*, Figs. S4 and S5). The absence of a Zn response may be potentially due to elevated in situ dZn concentrations (*SI Appendix*, Table S2) or the potential for metabolic substitution of some of the Zn requirement with Co or Cd in carbonic anhydrase (44, 45).

Overall, our experimental data were consistent with serial Fe–Mn–Si limitation developing as both the natural in situ bloom (Fig. 1) and artificially induced blooms within the experiments (Fig. 2) depleted ambient Mn, expanding the growing evidence of an important role for Mn availability in Southern Ocean productivity (4, 19, 32). Moreover, the spatiotemporal patterns observed in the experimental responses were broadly predictable on the basis of the relative availability of dMn and dFe compared with phytoplankton cellular requirements, as calculated using Mn_Fe_* (= dMn – dFe/R_FeMn_, where R_FeMn_, the assumed cellular molar stoichiometry of Fe:Mn, was taken as 2.67) (3, 4). Specifically, serial Fe–Mn responses were only observed in the low (negative) Mn_Fe_* (signifying Mn deficiency) waters to the south (Fig. 1), particularly following the peak of the bloom.

### Phytoplankton Responses to Patagonian Dust Addition.

Response to Patagonian dust addition revealed insights into the potential role of dust supply in Southern Ocean phytoplankton ecophysiology across naturally occurring biogeochemical gradients. For this study, dust samples included a subset of previously investigated glaciogenic (SMD13-3 and PMG) and nonglaciogenic (CAR19) sources from Patagonia (35). After both 2 and 6 d incubation, the addition of dust resulted in pronounced but varying responses in both *F_v_ /F_m_*, biomass accumulation, and nutrient drawdown, with the glaciogenic SMD13-3 generally providing a stronger response than either the other glaciogenic PMG or nonglaciogenic CAR19 sources (Figs. 2 and 3 and *SI Appendix*, Figs. S4 and S5).

Addition of all the dust sources significantly increased *F_v_ /F_m_* and chlorophyll-*a* after 2 d in all except one experiment (Station OOI Ex-S8) (*SI Appendix*, Figs. S4 and S5). Of the three dusts, SMD13-3 promoted the strongest responses, which were frequently comparable to the 2 nmol L^−1^ inorganic Fe addition from the same experiments (Figs. 2 and 3). The stronger response to SMD13-3 addition corresponded with this dust having the highest Fe content by mass (10% compared with 1.3 and 3.7% for PMG and CAR19, respectively, *SI Appendix*, Table S3), a significant fraction of which is comprised of Fe^2+^ silicates (35). Hence, Fe mineralogy and solubility likely contributed to the stronger short-term (2-d) experimental responses, as serial Mn responses were less apparent over these timescales (Fig. 2).

After 6 d, the addition of SMD13-3 to station OOI Ex-L5 increased *F_v_ /F_m_* (Fig. 2) to values comparable to the Fe additions, with no apparent serial Mn response. In contrast, the response to SMD13-3 addition in station TN Ex-L7 was elevated above that of Fe(±Si) treatments and equal in magnitude to the combined FeMn(±Si) additions. Moreover, unlike in the Fe(±Si) treatments, no drop in *F_v_ /F_m_* from 2 to 6 d was observed in the SMD13-3 dust treatment for TN Ex-L7. These experimental biotic responses thus indicated that Mn released from SMD13-3 particles could alleviate the observed serial Fe–Mn limitation of photochemical energy conversion. Similarly, and in contrast with previous responses of a cultured diatom grown under Fe-deplete, Mn-replete conditions (35), the photophysiological response to the glaciogenic PMG dust source in station TN Ex-L7 was lower relative to the nonglaciogenic CAR19 source. Dust addition also stimulated chlorophyll-*a* accumulation (Fig. 2) and DIN drawdown after 6 d in a similar fashion to that observed for *F_v_ /F_m_*, with differences in response again related to dust source. Specifically, the SMD13-3 dust provided the largest change in biomass with lower responses to CAR19 and PMG additions. The change in biomass was either comparable or less than that from Fe addition in all experiments. Decreasing biotic responses to dust additions (SMD13-3 >> CAR19 > PMG), in particular within the serial Fe–Mn-limited Ex-L7 experiment (Fig. 2 *E* and *F*), thus indicated variable amounts and/or kinetics of release of both Fe and Mn.

### Differential Phytoplankton Responses Linked to Source Region Dust Characteristics.

**Fig. 4.**
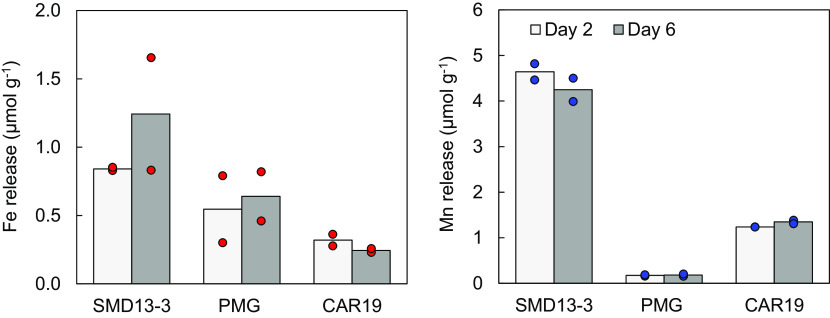
Patagonian dust dissolution. Mean (± 1 SD) of dissolved (0.2 µm) trace metal concentrations per unit mass added resulting from leaching of glaciogenic (SMD13-3, PMG) and nonglaciogenic (CAR19) Patagonian dusts into ambient Southern Ocean seawater. Colored circles represent experimental duplicates.

To assess how Fe and Mn were released into seawater and how this may have driven the observed biotic responses, we determined total element contents of the different dust sources and conducted controlled abiotic leaching experiments designed to simulate the shipboard bottle experiments as closely as possible. Measurable dissolved Fe and Mn was leached from each dust source by the 2-d time point (Fig. 4). No further significant increase in measurable dFe or dMn occurred between days 2 and 6 (Fig. 4), consistent with the majority of labile Fe and Mn mobilizing rapidly following contact with seawater (46–48). Typical crustal Mn/Fe ratios of less than 0.02 mol mol^−1^ are expected to be partially offset by fractional Mn solubility in atmospheric aerosols being around one order of magnitude greater than for Fe (47, 49–51), due to much of the Fe in mineral particles being located within refractory phases (52), combined with leached Fe^2+^ rapidly oxidizing to Fe^3+^ under oxygenic conditions. Consistent with this, fractional solubilities within our experimental setup for Fe and Mn were around 0.23 and 1.79%, respectively, for the glaciogenic PMG source (*SI Appendix*, Table S3), but were even more extreme for the other two sources, with values of 0.05% (Fe) and 7.29% (Mn) for the glaciogenic SMD13-3 source and 0.05% (Fe) and 8.82% (Mn) for the nonglaciogenic CAR19 source. The SMD13-3 and PMG glaciogenic dust sources used in this study were more highly reduced (35), which, in addition to providing soluble Fe^2+^, could also provide highly soluble reduced Mn^2+^ species. However, the reason for the higher apparent Mn solubility for nonglaciogenic CAR19 is less clear.

Consistent with the SMD13-3 dust driving the strongest biotic experimental responses (Fig. 2), it was the strongest source of both leachable dissolved Fe and Mn, releasing 0.84 and 4.64 µmol g^−1^, respectively (Fig. 4), equating to dissolved additions of 0.21 and 0.42 nmol L^−1^ dFe and 1.16 and 2.32 nmol L^−1^ dMn to our large- and small-volume experiments, respectively. Addition of dFe from SMD13-3 to Ex-L7 at station TN was thus one order of magnitude lower than that of the inorganic Fe addition, providing further support for the elevated *F_v_ /F_m_* compared with that of Fe after 6 d incubation being due to simultaneous Mn release (Fig. 2 *E* and *F*). Indeed, the dissolved Mn/Fe leach ratio of 5.52 mol mol^−1^ for SMD13-3 would have provided an excess of Mn relative to Fe and biotic requirements (3, 4, 53), with an estimated Mn_Fe_* increase of 1.03 ± 0.09 (1 SD) nmol L^−1^.

A similar dissolved Mn/Fe leach ratio of 3.86 mol mol^−1^ was observed for the nonglaciogenic CAR19; however, the lower concentrations of both Fe and Mn released would have produced an Mn_Fe_* of 0.31 ± 0.02 nmol L^−1^. In contrast, glaciogenic PMG released more Fe than Mn with an Mn/Fe leach ratio of 0.32 mol mol^−1^ corresponding to an Mn_Fe_* of 0.00 ± 0.03 nmol L^−1^. The relative release of Fe and Mn from these natural dust sources was thus decoupled from the total mass content, due to highly variable solubilities (*SI Appendix*, Table S3), with the relative strengths of the biotic responses, particularly after 6 d (Figs. 2 and 3) SMD13-3 >> CAR19 > PMG, being relatable to the same sequence of higher absolute Fe and Mn additions and higher Mn_Fe_* (Fig. 4).

Previous laboratory experiments that observed stronger responses to SMD13-3 and PMG than CAR19 under Mn-replete, Fe-deficient conditions were largely driven by the higher Fe^2+^ content of the physically weathered glaciogenic sources (35, 38). However, within the Mn-deficient (Mn_Fe_* < ≈ 0) waters of the southeast Pacific SAZ (Fig. 1*H*), the enhanced Mn supply from the glaciogenic SMD13-3 and nonglaciogenic CAR19 drove the stronger response, highlighting the importance of both in situ nutritional status of the community and the multi-trace-metal characteristics of any inputs in determining system responses.

### Extrapolating Experimental Responses to Wider Scales.

Care must clearly be taken in extrapolating experimental responses such as those presented here to natural system responses to similar drivers (i.e., dust inputs) over different time and space scales (3, 4, 54–56). Our experimental dust additions were of comparable magnitude to modern total annual inputs to the South Atlantic sector of the Southern Ocean, which are around two orders of magnitude higher than deposition to the study region (Fig. 1*A*). Moreover, these experiments were monitored over a 6-d time period in a closed system, which is very different to an in situ open system (54). Indeed, differences in the extent to which the strongest combined Fe and Mn dust source (SMD13-3) could fully reproduce the physiological (e.g., *F_v_ /F_m_*) and biomass (e.g., chlorophyll)-related biotic responses to direct FeMn(±Si) addition from 2 to 6 d were likely due to the order of magnitude lower Fe input from the dust, combined with progressive responses, as artificial blooms proceeded in the bottles. However, combined with other recent observations (4, 32), the progressive in situ development of Mn (co-)deficiency (Fig. 1*H*) and serial limitation (Fig. 1 *J* and *K*) during the bloom to the south of our study region and clear biotic responses to dust-related Mn inputs (Figs. 2 and 3) clearly argue for consideration of larger-scale biogeochemical consequences.

### Biogeochemical Implications.

The flux of mineral dust into Southern Ocean waters is among the lowest found on Earth (50). The present-day dust inputs to the Southern Ocean from South America, South Africa, and Australia follow the circumpolar flow of the westerly winds, predominantly fertilizing surface waters of the Atlantic, Indian, and Pacific sectors, respectively (40, 50, 57). The peak of dust deposition occurs during austral spring and summer (24, 39), contributing to the SAZ mixed-layer Fe and Mn inventories that already hold the potential for Fe-Mn (co-)limitation due to Mn deficiency relative to Fe, i.e., Mn_Fe_* ≤ 0 (4, 6).

For the Southeast Pacific sector of the SAZ, we observed near-zero or negative postbloom Mn_Fe_* values (Fig. 1*H*) similar to the Mn (co)-limited central Drake Passage (4). The source of dust, and by association mineralogy, is therefore likely to be an important influence on productivity in the SAZ. Specifically, the deposition of dust with high Mn/Fe leach ratios (e.g., SMD13-3, CAR19) would be expected to strengthen regional Fe limitation and/or prevent development of Mn (co-)limitation, while deposition of sources with lower Mn/Fe leach ratios (e.g., PMG) could strengthen Mn (co-)limitation, particularly toward the end of seasonal growth.

Dust-derived Fe to the SAZ has been associated with atmospheric CO_2_ drawdown during the last glacial cycle (9, 13). In contrast to modern/Holocene dust transport, South America was the dominant source of dust to the Pacific Southern Ocean during glacial periods (34). Such changes in source provenance could directly affect the relative magnitude of Fe and Mn delivery, with cascading effects on macronutrient utilization and CO_2_ drawdown. Indeed, reconstructions estimate a ~15-fold increase in Fe^2+^ supply to regions of the glacial Southern Ocean in comparison with interglacial periods, suggesting a role for glacier-derived Fe fertilization in the observed declines in atmospheric CO_2_ (41). However, our data suggest that dust may be significant in providing both Fe and Mn as biologically essential and potentially co-limiting micronutrients. The efficiency of dust-induced increases in glacial macronutrient utilization and hence ultimately atmospheric CO_2_ drawdown may thus have depended on source region influences on both Fe and Mn supply (4, 19).

Despite uncertainty as to how anthropogenic climate change will modify global dust deposition rates (16, 17), it has been proposed that the expansion of deserts and strengthening of winds could increase Southern Hemisphere atmospheric dust loading by the end of the 21st century (18). Ocean biogeochemical models also predict strengthening and poleward displacement of westerly winds over the 21st century (58–60), further influencing dust mobilization and source regions. Given the proximity to Southern Hemisphere dust source regions, any increase in deposition rates would likely be greatest between 40 and 60° S.

The net effect of altered dust flux on Southern Ocean biogeochemistry further depends on additional climate-related changes. The balance between intensification and poleward displacement of westerly winds acting to increase upwelling and subsurface nutrient supply versus warming and freshening of surface waters that can act to increase stratification (58, 61) should determine whether mixed-layer productivity increases or decreases (20, 62). The relative importance of dust-borne supply of Fe and Mn to the SAZ can thus be influenced by circulation-related changes in the subsurface entrainment flux of both macro- and micronutrients. For example, the deficiency of both Fe and Mn in upwelling circumpolar deep-waters (6) means that any regionally reduced subsurface supply of these scavenged micronutrients, potentially coupled with any increased atmospheric dust deposition, might be expected to result in a higher biological pump efficiency, as defined by macronutrient utilization (3, 15, 63). Changes in the magnitude of dust deposition, variations in source regions, and altered circulation patterns could hence all combine to determine whether Fe or Mn limitation controls productivity and biological CO_2_ uptake potential of the Southern Ocean under future as well as past climate states. However, ocean biogeochemical models currently typically only consider dust deposition from the perspective of Fe supply (9, 19, 64). Our results suggest that dust-borne Mn can have a significant influence on Southern Ocean phytoplankton ecophysiology, emphasizing the need for the incorporation of multi-element dust-driven fluxes in both past and future climate predictions.

## Materials and Methods

### Voyage and Seawater Collection.

Experiments and sample collection were carried out on the *R.R.S. Discovery* DY111 cruise (December 2, 2019 to January 9, 2020), a component of the UK Carbon Uptake and Seasonal Traits in Antarctic Remineralisation Depth (CUSTARD) project (Fig. 1). A total of fifteen nutrient and dust addition experiments (Fig. 1 and *SI Appendix*, Fig. S6) were conducted along the 89° W meridian at 54° S (Station OOI), 57° S (Station TN), and 60° S (Station TS).

### Nutrient Amendment Experiments.

Eight small-volume (2 to 6 d, denoted Ex-S1-S8) and seven large-volume (6 d, denoted Ex-L1-L7) factorial nutrient amendment experiments were conducted (*SI Appendix*, Fig. S6), using similar methods to those employed previously in the HNLC Southern Ocean and elsewhere (28–30, 42). Surface seawater (2 to 3 m) for experiments was pumped directly into a class-1000 clean air laboratory container using a Teflon diaphragm pump (A-15, Almatec) connected by acid-washed PVC tubing to a towed “Fish” sampler. For each experiment, seawater from the towed fish was collected into either 2 L (Ex-S) or 4 L (Ex-L) acid-washed polycarbonate bottles. Bottles were first filled in random order up to ~50% of total volume, before then being topped up, again in random order. Samples for initial measurements (photophysiology, chlorophyll-*a,* and macronutrients) were collected at the beginning, middle (i.e., after all experimental bottles were ~50% full), and end of the filling process. Separate replicate bottles were amended with single or combination additions of Fe, Mn, Zn, and Si to final concentrations of 2 nmol L^−1^ for each trace metal and 10 µmol L^−1^ for Si. Control bottles, with no added nutrients, were collected in parallel. Fe, Mn, and Zn were added as FeCl_3_, MnCl_2_, and ZnCl_2_, respectively, prepared from ≥99% purity salts (Sigma-Aldrich) and stabilized in 0.024 M HCl (SpA, Romil). The Si solution was prepared from Na_2_SiF_6_ salt (Sigma-Aldrich) and passed through a column of cation exchange resin (Chelex-100, BioRad) to remove trace metal impurities.

Three small-volume and two large-volume experiments were chosen to investigate the physiological response of resident phytoplankton communities to Patagonian dust amendment. Sediment samples were prepared and sterilized per the methods described by Shoenfelt et al. (35), with the current study also adopting the same sample names. Glaciogenic (SMD13-3 and PMG) and nonglaciogenic (CAR19) sediment was added to separate incubation bottles by triple rinsing (using filtered surface seawater) of the material from sterile 0.5-mL microcentrifuge vials and was accompanied by replicate controls whereby identical empty vials were rinsed into separate incubation bottles. No significant differences were observed between controls and these separate dust treatment controls (Fig. 2 and *SI Appendix*, Figs. S4 and S5). The addition of a fixed 1 mg mass of each dust source resulted in final additions of 0.5 and 0.25 mg L^−1^ for small- and large-volume experiments, respectively. All experimental incubations were conducted as biological triplicates, apart from Ex-S6 (all duplicates) and the dust additions to Ex-S8, Ex-L5, and Ex-L7 that were also duplicates. Following nutrient amendment, the bottles were externally sealed with film (Parafilm^TM^) and incubated in a temperature-controlled container set to local sea surface temperature (5.5 to 7.0 °C during the study) and surrounded by daylight simulation LED light banks with ~200 μmol photons m^−2^ s^−1^ flux and set to an approximate local day/night cycle of 16 and 8 h, respectively.

### Subsampling.

All experimental bottles in every experiment were subsampled for the same set of variables measured for initial conditions (photophysiology, chlorophyll-*a,* and macronutrients) after 48 h (2 d) in the clean air laboratory, at which point six of the eight small-volume experiments (Ex-S1-S3 and S6-S8) were terminated. To allow for any potentially slower photophysiological and growth response to Zn addition, Ex-S4 and Ex-S5 were allowed to incubate for a further 4 d at which point (i.e., day 6) they were sampled for a second and final time. All the large-volume experiments (Ex-L1-L7) were subsampled at 2 d and terminated after 6 d following a second and final sampling.

### Phytoplankton Photosynthetic Physiology.

Variable chlorophyll fluorescence was measured using a Chelsea Scientific Instruments Fastracka^TM^ Mk II Fast Repetition Rate fluorimeter (FRRf) integrated with a FastAct^TM^ laboratory system. Samples (~125 mL) were dark-acclimated for 30 min in opaque bottles (Nalgene), and FRRf measurements were blank corrected using carefully prepared 0.2 µm filtrates and 18.2 MΩ-cm ultrapure water for each experiment (28). Blanks contributed 2 ± 1% (*n* = 15) of the maximum fluorescence signal during this study. Protocols for FRRf measurements and data processing were similar to those detailed elsewhere (28). Data from the FRRf were analyzed to derive values of the minimum and maximum fluorescence (*F*_o_ and *F*_m_, respectively) and the apparent photochemical efficiency of photosystem II (PSII), *F*_v_ /*F*_m_ (where *F*_v_ = *F*_m_ − *F*_o_) (65). A phytoplankton response from the nutrient or dust amendment experiments was represented by a significant increase in *F*_v_/*F*_m_ over that of the control bottles.

### Chlorophyll.

Samples for chlorophyll-*a* analysis (~100 mL) were filtered onto 0.7 µm nominal porosity GF/F filters and extracted into 90% acetone for 24 h in the dark at 4 °C prior to measurement. Chlorophyll-*a* concentration was determined on a precalibrated Turner Designs Trilogy fluorometer (66) while at sea within 48 h of collection.

### Macronutrients.

The dissolved concentrations of macronutrients such as phosphate (PO_4_^3-^), silicate (SiO_2_; as Si hereafter), and nitrate (determined as nitrate + nitrite) were measured onboard ship within 24 h of sample collection using a SEAL QuAAtro 39 segmented flow autoanalyzer following colorimetric procedures provided by SEAL Analytical (Q-064-05, Q-066-05, Q-068-05). Certified Reference Materials (CRM Lots CJ and CB, KANSO, Japan) were used to check for accuracy of the method with excellent agreement.

### Trace Metals.

Seawater samples for ambient dissolved trace metal determination were collected from the towed “Fish” sampler during the “filling” stage of each experiment, alongside experimental initial samples. Seawater was filtered through 0.2 µm porosity membrane cartridge filters (Sartobran, Sartorius) into trace metal–clean 125 mL low-density polyethylene bottles and acidified to pH 1.7 (0.024 M) by addition of 12 M ultrapure HCl (UpA, Romil) under a class-100 laminar flow hood in the clean air container. Samples were double bagged in polyethylene bags and stored for analysis. Dissolved trace metal concentration was determined using a standard addition method (67, 68) with off-line preconcentration and subsequent high-resolution ICP-MS (69, 70) at the National Oceanographic Centre, Southampton, UK. Certified values for SAFe (S and D2) reference material compared well with our measured values (*SI Appendix*, Table S1).

### Dissolution Experiments.

Dust-leaching experiments were designed to replicate dust addition to the bioassay experiments as closely as possible. Leaching experiments were performed after the cruise inside a class-1000 clean air laboratory at the National Oceanographic Centre, Southampton, UK, under controlled physicochemical conditions (12 h light/dark cycles, ~25° C) using filtered Southern Ocean surface seawater collected from the “Fish” sampler during DY111 (−56.9° S, −88.7° E). Glaciogenic (SMD13-3 and PMG) and nonglaciogenic (CAR19) sediments were added to 1.5 L of seawater in 2 L acid-washed and well-rinsed polycarbonate bottles according to the methods outlined for the nutrient amendment experiments. Subsamples for dissolved trace metal analysis were taken after 2 and 6 d by filtration through acid-washed, 0.2 µm pore size, 25 mm polycarbonate track-etched membrane filters (Nuclepore, Whatman) before acidification (0.024 M) by addition of 12 M ultrapure HCl (UpA, Romil). The filtration manifold consisted of acid-cleaned PTFE manifold tubing and PVC peristaltic pump tubing. A new filter was used for each subsample. Dissolved trace metals in subsamples from the dissolution experiment were analyzed at the same time as for the DY111 ambient samples. While metal adsorption to bottle walls may occur over a 2-d period (71), this effect would have been minimized through the large surface area-to-volume ratio of our experiment bottles, while reasonably rapid establishment of equilibrium occurred, as we measured no significant change in either Fe or Mn between 2- and 6-d time points.

### Dust Particle Digestion.

Dust samples were fully digested after the cruise following recommended GEOTRACES protocols (72). Briefly, the samples were transferred into trace metal–clean perfluoroalkoxy vials (Savillex) under a laminar flow fume hood in a class-1000 clean laboratory, into which 1 mL of digest solution (50% HNO_3_ + 10% HF, v/v, Optima Grade, Romil) was pipetted. Vials were refluxed at 135 °C for 4 h and then evaporated to near dryness. Once cooled, 100 µL of concentrated HNO_3_ was added to each vial and the samples were dried down again. The samples were redissolved in 3 mL of 5% HNO_3_ solution (spiked with 1 ppb indium as a drift monitor) and refluxed at 135 °C for 1 h. A 0.5 mL aliquot of each sample was diluted to 3 mL with the same 5% HNO_3_ solution in trace metal–clean, polyethylene Omni-vials (DWK Wheaton^TM^), for analysis by HR-ICP-MS (Element XR—Thermo Scientific^TM^) at the National Oceanography Centre, Southampton, UK.

### Statistical Analysis of Experimental Results and Interpretation of “(co-)limitation”.

Differences between the various response variables within the experiments were assessed using ANOVA followed by a Bonferroni means comparison test (*P* < 0.05). We adopt a similar definition framework for different “types” of “(co-)limitation” as described previously (3, 42, 73), noting that evidence of both serial and independent responses frequently occurred, sometimes within the same experiment (e.g., Fig. 2 and *SI Appendix*, Figs. S1–S3). Moreover, we differentiate at points in the text between stress (a physiological response to environmental change, e.g., *F*_v_/*F*_m_, increasing following amendment with a nutrient indicating lower nutrient stress) and “limitation” (e.g., biomass or equivalently net growth rate increases following amendment with a nutrient indicating lowered nutrient limitation) (42). We note that although the former (i.e., a physiological response) is clearly a necessary precursor for the latter, they are not synonymous and are timescale dependent (3). However, all provide evidence for multi-nutrient influence on phytoplankton ecophysiology.

## Supplementary Material

Appendix 01 (PDF)

## Data Availability

All data and metadata from this article and/or *SI Appendix* are publicly available from the British Oceanographic data Centre (https://www.bodc.ac.uk/data/published_data_library/catalogue/10.5285/fe06072e-d4c4-2201-e053-6c86abc067de/) (74).
