# Supplementary material for: Phytoplankton responses to dust addition in the Fe–Mn co-limited eastern Pacific sub-Antarctic differ by source region
Source: Proc Natl Acad Sci U S A. 2023 Jul 3;120(28):e2220111120. doi: 10.1073/pnas.2220111120 (PMC10334728; doi:10.1073/pnas.2220111120)
Supplement: Supplementary file 1 — Appendix 01 (PDF) [file pnas.2220111120.sapp.pdf]

Supporting Information for

Phytoplankton responses to dust addition in the FeMn co-limited eastern Pacific sub-Antarctic differ by source region.

Neil J. Wyatt, Antony Birchill, Simon Ussher, Angela Milne, Heather A. Bouman, Elizabeth Shoenfelt Troein, Katsiaryna Pabortsava, Alan Wright, Oliver Flannigan, Thomas S. Bibby, Adrian Martin, C. Mark Moore.

Neil J. Wyatt

Email: neil.j.wyatt@plymouth.ac.uk

This PDF file includes:

Tables S1 to S3

Figures S1 to S6

Table S1. **Trace metal intercalibration data.**

	SAFe measured (nmol L^{-1})		SAFe consensus (nmol L^{-1})		Detection limit (nmol L^{-1})	RSD (%)
	S	D2	S	D2		
Iron	0.093±0.012	1.164±0.068	0.096±0.008	0.959±0.024	0.035	<13
Manganese	0.768±0.062	0.365±0.017	0.812±0.062	0.360±0.051	0.051	<8
Zinc	0.067±0.012	7.272±0.391	0.071±0.010	7.634±0.257	0.036	<18

Accuracy of the analytical method was validated by repeat quantification of dissolved Fe, Mn, and Zn in SAFe reference seawater. Detection limits were calculated as 3x1σ of the lowest concentration reference sample and Relative Standard Deviation (RSD) from the mean (n=3) and 1σ of the lowest concentration reference sample.

Table S2. **Experiment starting conditions.**

Exp.	Lat.	Lon.	DIN	Phosphate	Silicate	Fe	Mn	Zn	Chl-*a*	F_v/F_m
OOI Ex-S1	-54.01	-85.34	20.318	1.374	5.113	0.037	0.222	0.448	0.486	0.284
OOI Ex-S2	-54.56	-89.14	19.950	1.340	4.535	0.035	0.214	0.346	0.377	0.260
TN Ex-S3	-57.31	-89.22	19.517	1.316	3.783	0.041	0.193	1.863*	0.762	0.221
TN Ex-S4	-57.61	-88.76	16.823	1.041	0.047	0.056	0.051	0.189	2.416	0.196
TN Ex-S5	-57.39	-90.06	17.013	1.068	0.077	0.017	0.025	0.143	1.108	0.207
Ex-S6	-58.89	-89.14	18.460	1.176	0.053	0.084	0.034	0.189	0.757	0.179
Ex-S7	-58.16	-90.62	18.317	1.162	0.143	0.029	0.025	0.160	0.763	0.172
OOI Ex-S8	-54.54	-89.13	18.600	1.195	2.220	0.040	0.122	0.161	0.461	0.145
OOI Ex-L1	-54.39	-88.53	20.220	1.364	4.843	0.096	0.169	0.267	0.377	0.189
TS Ex-L2	-59.98	-89.26	24.117	1.532	7.063	0.032	0.100	0.769	1.702	0.167
OOI Ex-L3	-54.61	-89.01	19.623	1.321	3.833	0.064	0.182	0.247	0.506	0.164
TS Ex-L4	-59.84	-89.37	21.243	1.334	1.013	0.055	0.037	0.658	2.596	0.164
OOI Ex-L5	-54.48	-89.03	19.130	1.277	3.427	0.047	0.147	0.189	0.591	0.162
TS Ex-L6	-59.91	-89.49	23.093	1.458	0.727	0.050	0.022	0.342	1.031	0.152
TN Ex-L7	-57.10	-89.12	17.143	1.100	0.097	0.020	0.026	0.088	0.796	0.142

Units: Latitude, °N; Longitude, °E; Macronutrients (DIN, phosphate, silicate), µmol L^{-1}; Dissolved trace metals (Fe, Mn, Zn), nmol L^{-1}; Chl-*a*, µg L^{-1}; F_v/F_m, unitless. Macronutrient, Chl-*a* and F_v/F_m values represent an average calculated from the measurement of triplicate initial samples. Trace metal data represent the mean of triplicate measurement on the same sample. Here DIN represents nitrate + nitrite. *Sample likely contaminated.

Table S3. **Total and soluble Fe and Mn in Patagonian dust sources**.

Dust source	Source origin	Total concentration (μmol g^{-1})		Fractional mass content (%)		Fractional solubility (%)	
		Fe	Mn	Fe	Mn	Fe	Mn
SMD13-3	Glaciogenic	1793±31	64±1.0	10.01±0.17	0.350±0.005	0.05±0.00	7.29±0.40
PMG	Glaciogenic	235±5	10±0.1	1.31±0.03	0.053±0.001	0.23±0.15	1.79±0.20
CAR19	Non-glaciogenic	671±16	14±0.1	3.75±0.09	0.077±0.001	0.05±0.01	8.82±0.02

Total concentration and mass content following total particle digestion. Fractional solubility following 2-day seawater leach into ambient Southern Ocean seawater. Concentration error represents 1σ of repeat analysis (n=3) whilst solubility error represents 1σ of dissolution experiment duplicates.

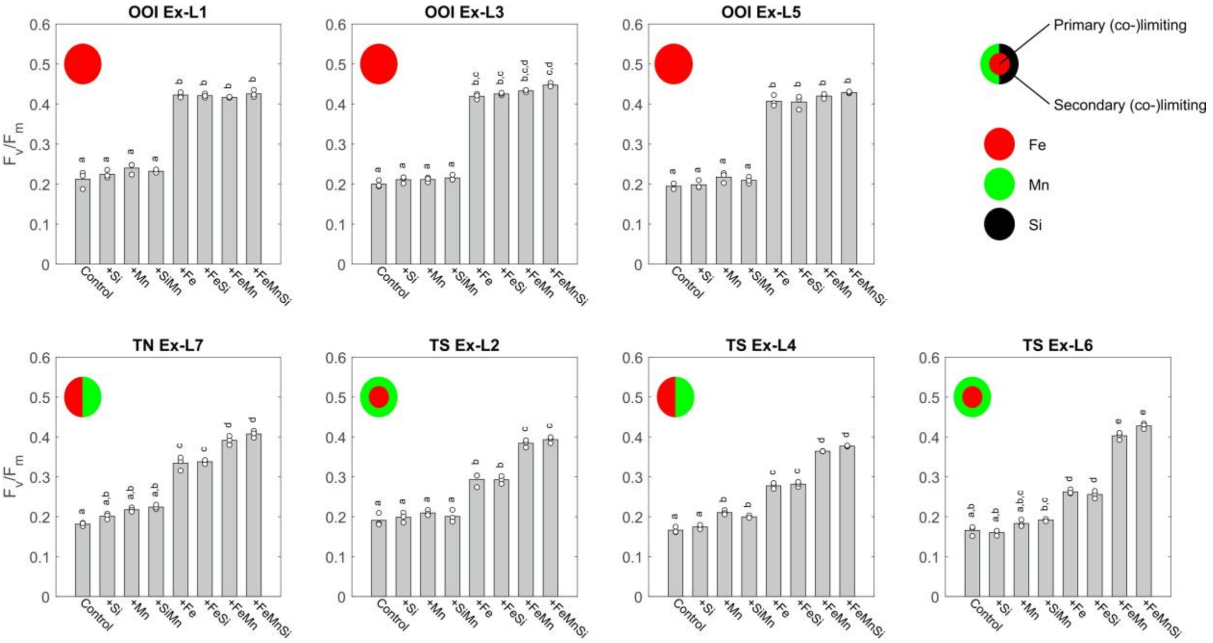

Figure S1. **Apparent photochemical efficiency of PSII (F_v/F_m) from larger volume experiments at 6 days**. Mean values (bars) with individual data points (small symbols) are shown. Statistically indistinguishable means evaluated across all treatments in full factorial manner are labelled with the same letter (analysis of variance (ANOVA) followed by Bonferroni post-hoc means comparison test $P \leq 0.05$). Large round symbol colors indicate the identity and type of limitation diagnosed from the statistical responses.

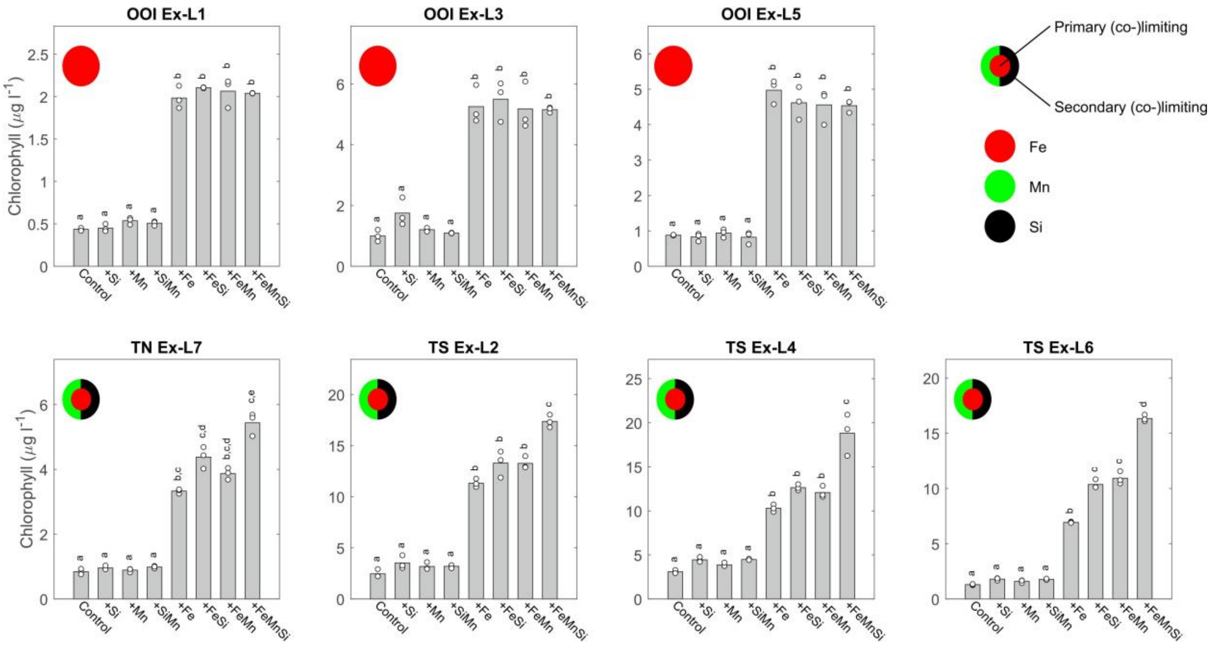

Figure S2. **Chlorophyll-*a* from larger volume experiments at 6 days**. Mean values (bars) with individual data points (small symbols) are shown. Statistically indistinguishable means evaluated across all treatments in full factorial manner are labelled with the same letter (analysis of variance (ANOVA) followed by Bonferroni post-hoc means comparison test $P \leq 0.05$). Large round symbol colors indicate the identity and type of limitation diagnosed from the statistical responses.

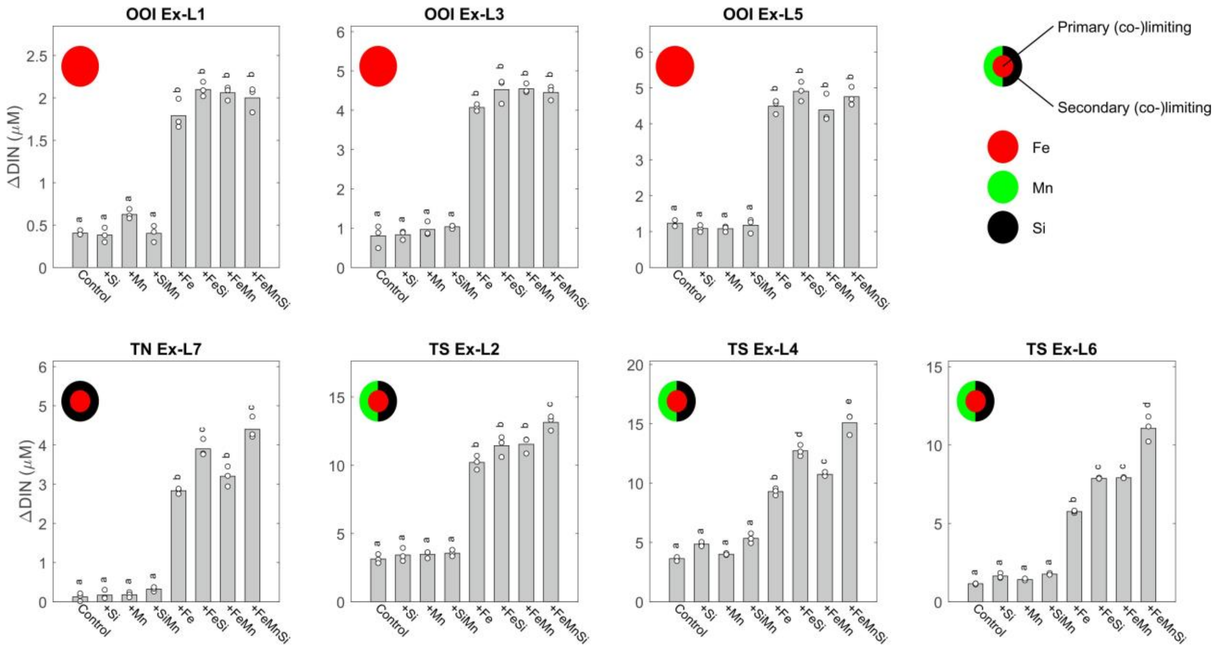

Figure S3. **Dissolved inorganic nitrogen drawdown from larger volume experiments at 6 days**. Mean values (bars) with individual data points (small symbols) are shown. Statistically indistinguishable means evaluated across all treatments in full factorial manner are labelled with the same letter (analysis of variance (ANOVA) followed by Bonferroni post-hoc means comparison test $P \leq 0.05$). Large round symbol colors indicate the identity and type of limitation diagnosed from the statistical responses.

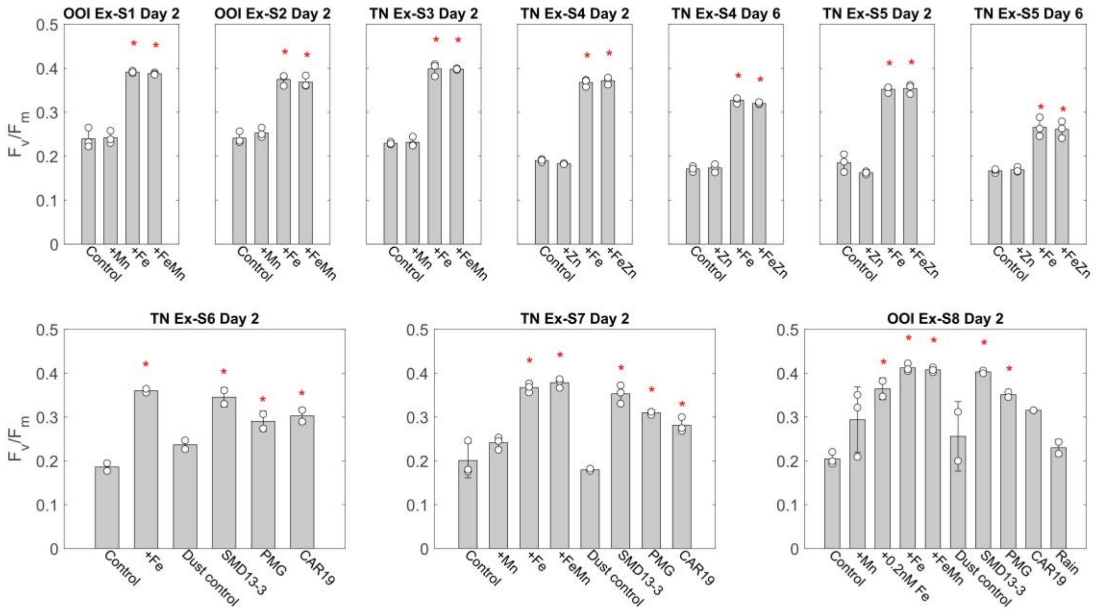

Figure S4. **Apparent photochemical efficiency of PSII (F_v/F_m) from smaller volume experiments**. For clarity, only the partial results of the statistical testing are included. Mean values (bars) with individual data points (small symbols) are shown. Error bars represent ± 1 standard deviation. Treatments that are statistically different from controls (ANOVA followed by Bonferroni means comparison test, $p < 0.05$) are labelled with a red asterisk.

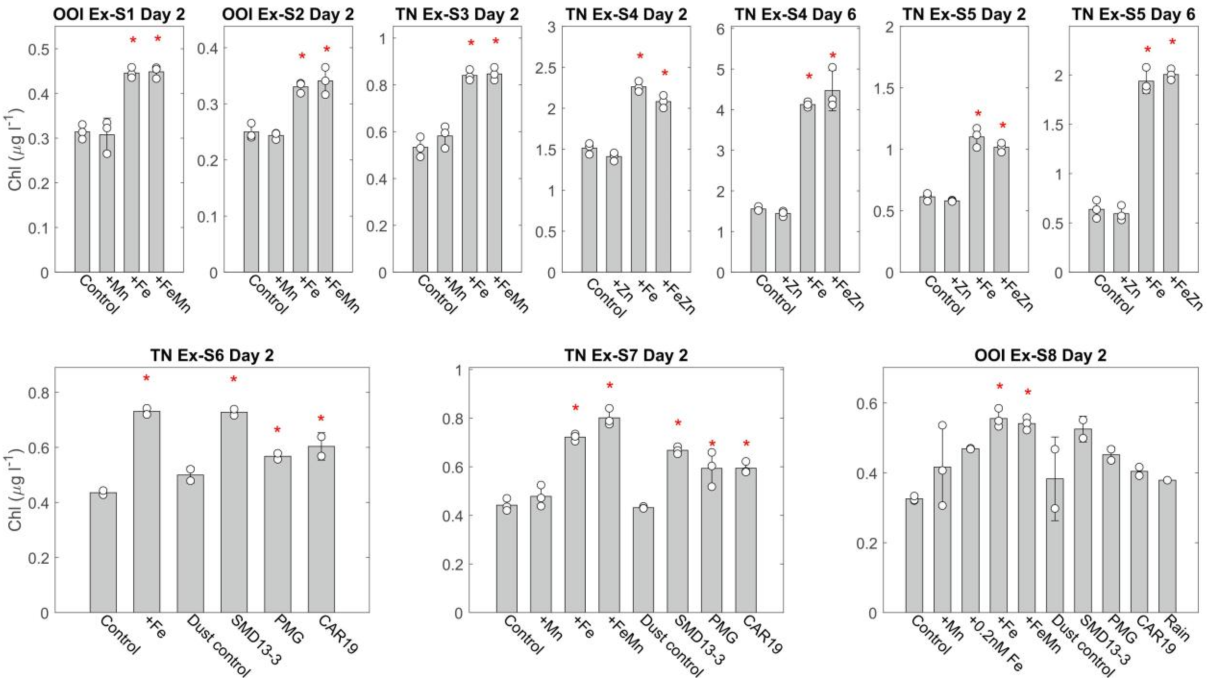

Figure S5. **Chlorophyll-*a* from smaller volume experiments**. For clarity, only the partial results of the statistical testing are included. Mean values (bars) with individual data points (small symbols) are shown. Error bars represent ± 1 standard deviation. Treatments that are statistically different from controls (ANOVA followed by Bonferroni means comparison test, $p < 0.05$) are labelled with a red asterisk.

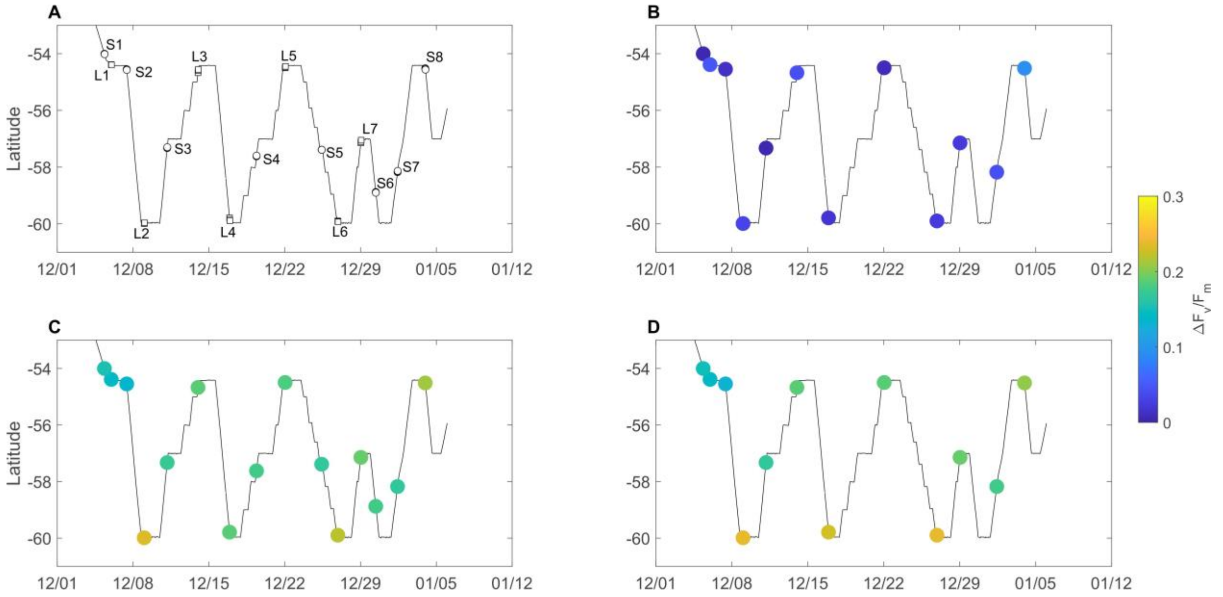

Figure S6. **Time and space variability in 48 h (2-day) responses of $\Delta F_v/F_m$ across all experiments**. (A) Experiment latitudes and dates superimposed on cruise track. (B) Response of F_v/F_m to amendment with Mn. (C) Response of F_v/F_m to amendment with Fe. (D) Response of F_v/F_m to amendment with Fe and Mn. Delta notation (Δ) indicates change relative to the value from control bottles.
